# Acetaminophen Induces Apoptosis in Rat Cortical Neurons

**DOI:** 10.1371/journal.pone.0015360

**Published:** 2010-12-10

**Authors:** Inmaculada Posadas, Pablo Santos, Almudena Blanco, Maríangeles Muñoz-Fernández, Valentín Ceña

**Affiliations:** 1 Unidad Asociada Neurodeath, CSIC-Universidad de Castilla-La Mancha, Departamento de Ciencias Médicas, Albacete, Spain; 2 Laboratorio de Inmunobiología Molecular, Hospital General Universitario Gregorio Marañón, Madrid, Spain; 3 CIBER de Enfermedades Neurodegenerativas, Instituto de Salud Carlos III, Madrid, Spain; 4 CIBER de Bioingeniería, Biomateriales y Nanomedicina, Instituto de Salud Carlos III, Madrid, Spain; Health Canada, Canada

## Abstract

**Background:**

Acetaminophen (AAP) is widely prescribed for treatment of mild pain and fever in western countries. It is generally considered a safe drug and the most frequently reported adverse effect associated with acetaminophen is hepatotoxicity, which generally occurs after acute overdose. During AAP overdose, encephalopathy might develop and contribute to morbidity and mortality. Our hypothesis is that AAP causes direct neuronal toxicity contributing to the general AAP toxicity syndrome.

**Methodology/Principal Findings:**

We report that AAP causes direct toxicity on rat cortical neurons both *in vitro* and *in vivo* as measured by LDH release. We have found that AAP causes concentration-dependent neuronal death *in vitro* at concentrations (1 and 2 mM) that are reached in human plasma during AAP overdose, and that are also reached in the cerebrospinal fluid of rats for 3 hours following i.p injection of AAP doses (250 and 500 mg/Kg) that are below those required to induce acute hepatic failure in rats. AAP also increases both neuronal cytochrome P450 isoform CYP2E1 enzymatic activity and protein levels as determined by Western blot, leading to neuronal death through mitochondrial–mediated mechanisms that involve cytochrome c release and caspase 3 activation. In addition, *in vivo* experiments show that i.p. AAP (250 and 500 mg/Kg) injection induces neuronal death in the rat cortex as measured by TUNEL, validating the *in vitro* data.

**Conclusions/Significance:**

The data presented here establish, for the first time, a direct neurotoxic action by AAP both *in vivo* and *in vitro* in rats at doses below those required to produce hepatotoxicity and suggest that this neurotoxicity might be involved in the general toxic syndrome observed during patient APP overdose and, possibly, also when AAP doses in the upper dosing schedule are used, especially if other risk factors (moderate drinking, fasting, nutritional impairment) are present.

## Introduction

Acetaminophen (paracetamol; AAP) is considered a non-steroidal anti-inflammatory (NSAID) drug, even though in clinical practice and in animal models it shows little anti-inflammatory activity [1]. However, like NSAIDs, AAP is used to treat pain and fever and it has become one of the most popular ‘over-the-counter’ non-narcotic analgesic agents. For instance, this compound has been taken, at least once, by more than 85% of children under the age of 91 months in the UK [2]. In the US, about 79% of the general population regularly takes AAP [3], including more than 35% of pregnant women [2]. The most frequently reported adverse effect associated with AAP is hepatotoxicity, which occurs after acute over dosage (usually doses greater than 10 g are needed) [4] and, less frequently, during long term treatment with doses at the higher levels of the therapeutic range [5] or in the presence of precipitating factors like fasting, nutritional impairment or alcohol intake [4]. Besides hepatic toxicity, no AAP toxic actions have been described in the nervous system, although it is well known that AAP crosses the blood-brain barrier both in rodents and humans [6]. Acetaminophen is mainly metabolised in the liver via conjugation with glucuronic acid and sulphate and then excreted, but, a small fraction is metabolised by cytochrome P-450 [7], [8] forming a chemically reactive metabolite, n-acetyl-p-benzoquinone imine (NAPQI), which reacts with GSH to form a non-toxic conjugate that will be excreted. Once GSH is exhausted, NAPQI binds to cellular proteins, including mitochondrial proteins, leading to hepatocellular death [9]. It has also been described that CYP2E1 is also expressed in the brain [10], suggesting that AAP might be metabolised by neurons producing the toxic metabolite NAPQI, which would lead to neurotoxicity. Although there is a previous report indicating that AAP potentiates staurosporine-mediated toxicity in neuroblastoma [11], information on direct AAP neurotoxicity has not been described.

Mitochondria play a key role in regulating the apoptotic mechanisms and also in some forms of cell death by necrosis [12], [13]. Calcium overload or free radical production induce the mitochondrial inner membrane permeabilization (MIMP) that promotes mitochondrial swelling, outer membrane rupture and release of intermembrane proapoptotic proteins such as cytochrome C (cyt C) and apoptosis inducing factor (AIF) to the cytoplasm [14]. These factors also activate caspases and, subsequently, caspase-activated DNase [15].

In this study, we have studied the effect of AAP on rat cortical neurons in culture and report, for the first time, that this widely used drug has a low but persistent toxicity on neurons through a mitochondrial-dependent mechanism involving cyt C release and caspase 3 activation. In addition, *in vivo* experiments in rats show that CSF levels achieved following i.p. AAP injection are similar to those drug concentrations that cause neuronal death *in vitro* and also produce a time-dependent neuronal death *in vivo* as measured by an increase in the number of TUNEL positive cells in the cortex. These data suggest that neuronal toxicity might be produced by APP, in addition to the well-known hepatic toxicity, and that it might contribute to AAP overdose toxicity.

## Results

### AAP induces apoptotic neuronal death

**Figure 1:**
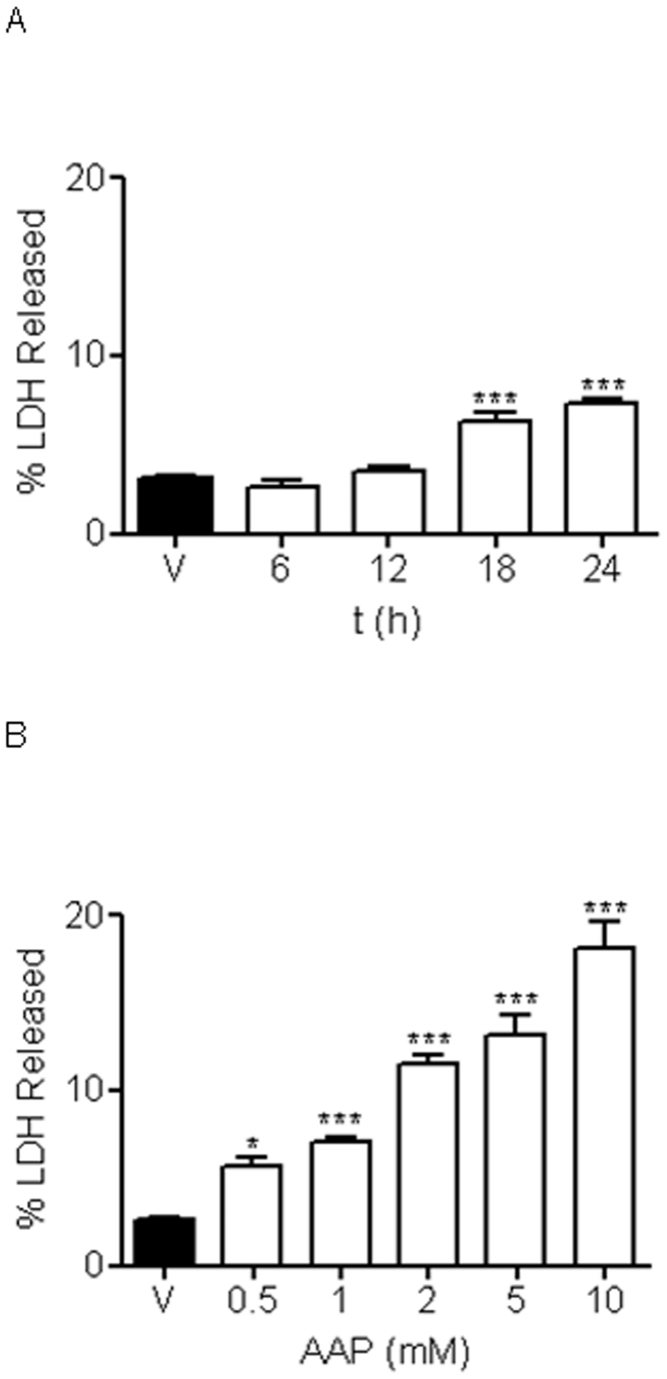
AAP reduces rat cortical neuron viability. (**A**) Time course effect of AAP treatment (1 mM) on cellular viability expressed as percentage of LDH released to culture medium. V stands for vehicle (DMSO 1‰)-treated cells. Data represent mean ± SEM of 12 experiments. ***p<0.01, as compared to vehicle-treated cells. (**B**) Concentration-response effect of AAP on rat cortical neuron viability 24 h after treatment. V stands for vehicle (DMSO 1‰)-treated cells. Data represent mean ± SEM of 12 experiments. *p<0.05 and ***p<0.01, as compared to vehicle-treated cells.

**Figure 2:**
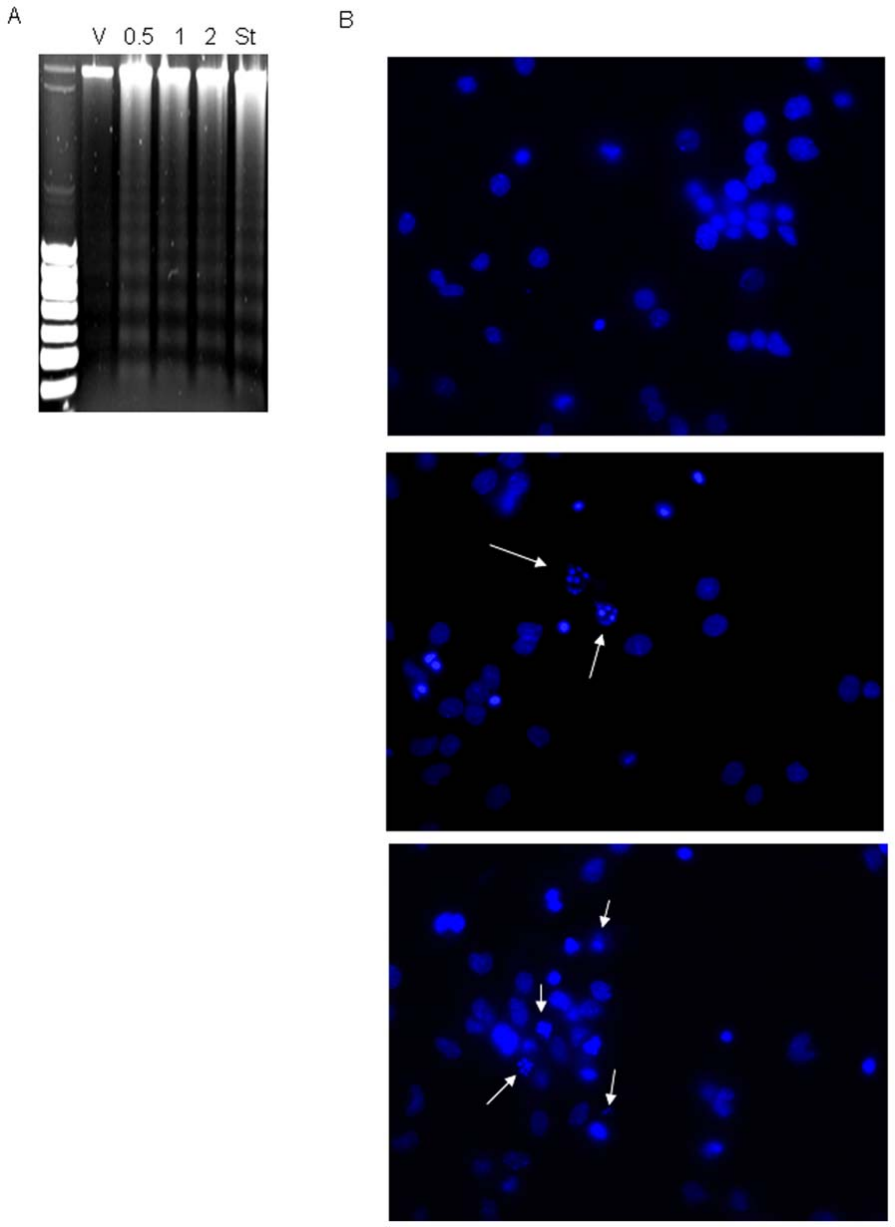
AAP induces apoptotic death in rat cortical neurons. (**A**) DNA degradation laddering pattern observed in rat cortical neurons after treatment with AAP 0.5, 1 and 2 mM or staurosporine (St), used as a positive control, for 24 h. Vehicle-treated cells (DMSO 1‰; V) did not show DNA degradation. (B) Hoechst 33342 staining of cortical neuron nuclei treated with vehicle (DMSO 1‰; upper panel), AAP 0.5 mM (middle panel) or AAP 1 mM (lower panel) for 24 h. Arrows show fragmented chromatin. Images are representative of one experiment repeated 3 times with similar results.

To test the effect of AAP on rat cortical neurons viability, cells were plated on poly-L-lysine-coated 24-culture plates and, after 7-10 DIV, incubated with different AAP concentrations for 24 h or with AAP 1 mM for 6, 12, 18 and 24 h. After the incubation period, supernatants and cell lysates were collected and LDH activity was measured. Cellular mortality was expressed as the percentage of LDH released to culture medium. AAP induced an increase in the percentage of LDH released that was time- and concentration-dependent (Figures 1A and 1B). For the next *in vitro* experiments, concentrations of AAP from 0.5 to 2 mM were chosen. Neurons died either by apoptosis, necrosis or a mixture of both types of death. To determine the type of AAP-induced neuronal death, DNA fragmentation was studied. DNA from vehicle-, staurosporine- (a well-known inducer of apoptosis used as internal control) and AAP-treated rat cortical neurons was extracted and samples were subjected to electrophoresis on agarose gel. After staining with ethidium bromide, observation under UV light showed the classical laddering pattern in AAP- and staurosporine-treated cells, but not in vehicle-treated cells (Figure 2A). This is consistent with AAP inducing apoptotic death in these neurons. In another set of experiments, rat cortical neurons (7-10 DIV) treated with AAP 0.5 and 1 mM for 24 hours showed chromatin condensation and nuclear fragmentation, a feature of apoptosis, whereas only round blue nuclei were observed in vehicle-treated cells (Figure 2B).

### Mitochondrial role in AAP-induced neuronal death

**Figure 3:**
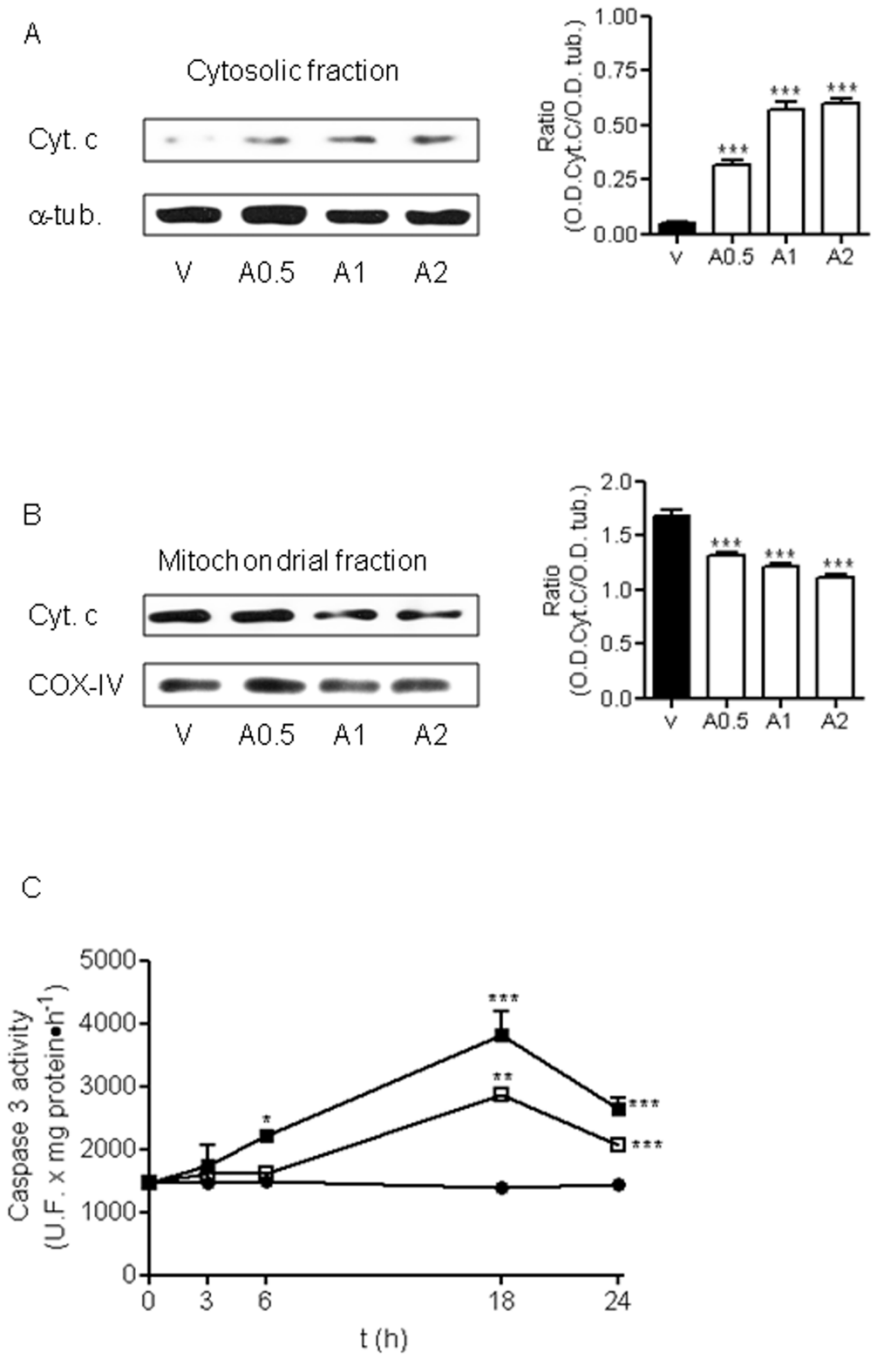
Lost of neuronal cells induced by AAP is mediated by activation of intrinsic apoptotic pathway. (**A**) Left. Cytochrome c (Cyt c) release from mitochondria to cytosol 24 h after AAP-treatment. Cells were treated with vehicle (DMSO 1‰; V) or AAP at 0.5 (A0.5), 1(A1) or 2 (A2) mM for 24 h and the cytosolic fraction was obtained. α-Tubulin (α-tub) was used as cytosolic protein loading control. Right. Densitometric analysis of Cyt c related to α-tubulin (α-tub.) protein levels detected in cytosolic fraction. Data are expressed as mean ± SEM of 3 independent experiments. ***p<0.001 as compared to vehicle-treated cells. (**B**) Left. Same experiment as in (**A**)**,** but Cyt c mitochondrial content was determined. COX-IV was used as mitochondrial protein loading control. Right. Densitometric analysis of Cyt c related to COX-IV protein levels detected in mitochondrial fraction. Data are expressed as mean ± SEM of 3 experiments. ***p<0.001 compared to vehicle-treated cells. (**C**) Time-course of caspase 3 activaty induced by AAP. Cortical neurons were incubated with vehicle (DMSO 1‰; •), AAP 1 mM (□) or AAP 2 mM (▪). After different time periods, cell lysates were obtained and caspase 3 activity determined as indicated in Material and Methods. Data represent mean ± SEM of 12 independent experiments. *p<0.05; **p<0.01; *** p<0.001 as compared to vehicle-treated cells. When not shown, SE bars were smaller than the symbol size.

**Figure 4:**
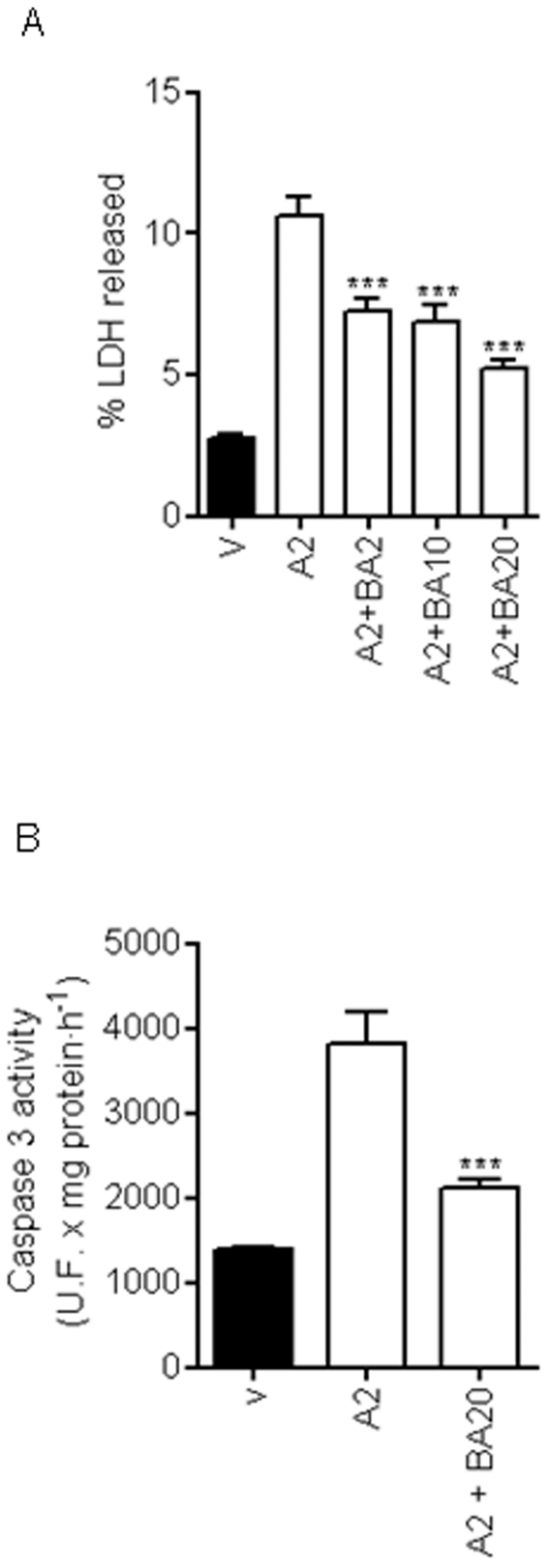
Bongkrekic acid (BA) prevents AAP-induced neuronal death. (**A**) BA, by inhibiting mitochondrial permeability transition, markedly reduced AAP-mediated toxicity on rat cortical neurons in a concentration-dependent manner. Cells were treated with vehicle (DMSO 1‰; V) or AAP 2 mM (A2) in the presence or absence of BA at different concentrations for 24 h and the percentage of LDH released to culture medium was quantified. Data represent mean ± SEM of 9 independent experiments. *** p<0.001 as compared to A2-treated cells. (**B**) BA prevents caspase 3 activation induced by AAP. Cells were treated with vehicle (DMSO 1‰; V) or AAP 2 mM (A2) in the presence or absence of BA 20 µM (A2+ BA20) for 18 h and caspase 3 activity in total lysates was determined. Data represent mean ± SEM of 12 independent experiments. *** p<0.001 as compared to A2-treated cells.

A role for mitochondria in AAP-mediated hepatic toxicity has been proposed [9], [16], so we explored whether AAP was able to induce the release of cyt C from this organelle in rat cortical neurons. As is shown in Fig. 3A, AAP at the concentration range used (0.5 to 2 mM) was able to induce cyt C release from mitochondria to neuronal cytosol that was accompanied by a decrease in mitochondrial cyt C content (Figs. 3A, 3B). Caspase-3 activity, as executioner protease of neuronal death, was studied [17], [18]. Acetaminophen treatment of rat cortical neurons in culture for 3, 6, 18 and 24 h induced caspase-3 activation that reached maximum levels at 18 h after AAP treatment, decreasing thereafter (Figure 3C). Moreover, pre-treatment of cortical neurons with the mitochondria permeability transition blocker bongkrekic acid (2 to 20 µM) markedly decreased both AAP-induced toxicity (Figure 4A) and caspase 3 activation (Figure 4B). Considering all these data as a whole indicates that AAP induces apoptosis in rat cortical neurons in culture and that mitochondria play a prominent role in this death.

### Acetaminophen induces neuronal death through free radical production

**Figure 5:**
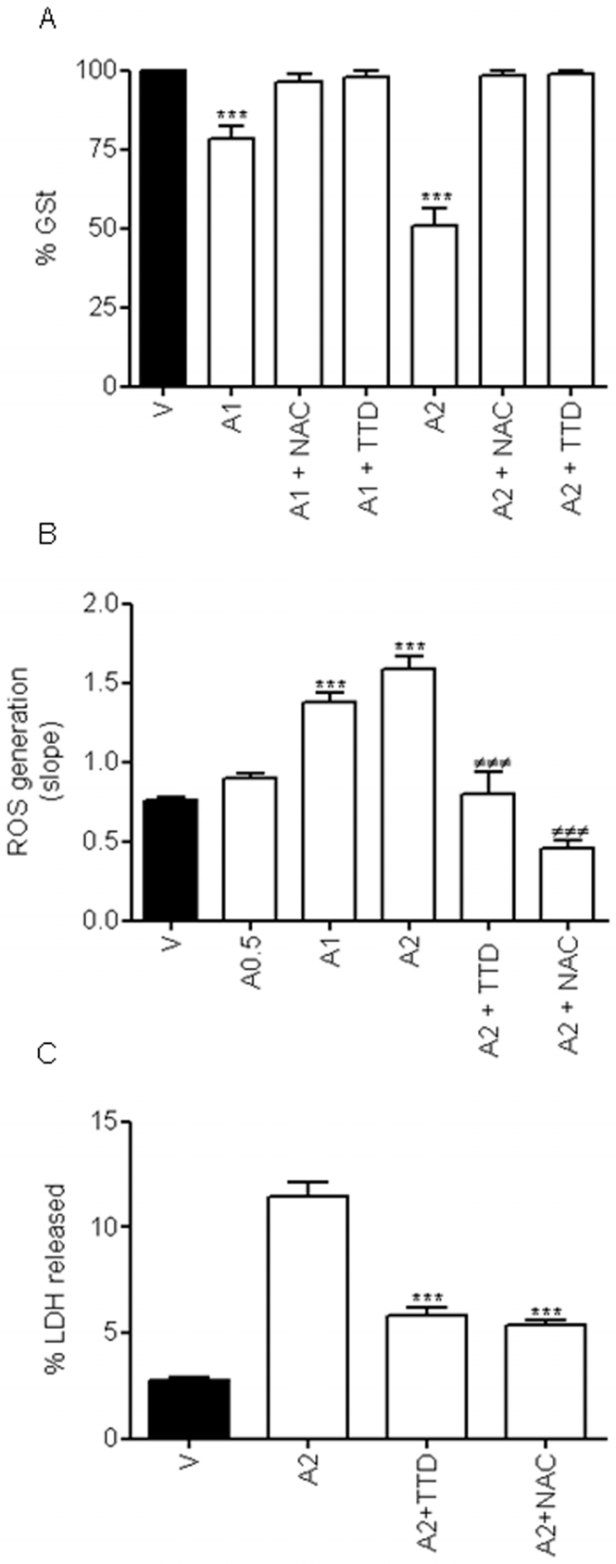
Redox status of rat cortical neurons. (**A**) Neuronal glutathione levels were decreased by treatment with 1 mM (A1) or 2 mM (A2) AAP in a concentration-dependent manner whereas co-treatment with either N-acetylcysteine (NAC; 100* µ*M) or disulfiram (TTD; 0.1 µM) completely restored glutathione content to basal levels. V stands for vehicle (DMSO 1‰)-treated cells. Data represent mean ± SEM of 9 independent experiments. *** p<0.001 as compared to vehicle-treated cells). (**B**) AAP 0.5 (A0.5), 1 (A1) and 2 mM (A2) induced a dose-dependent increase in the rate of reactive oxygen species (ROS) production, that was prevented by both N-acetylcysteine (NAC; 100* µ*M) and disulfiram (TTD, 0.1* µ*M). V stands for vehicle (DMSO 1‰)-treated cells. Data represent the mean ± SEM of 9 independent experiments. ***, p*<*0.001 as compared to vehicle-treated cells (V); ^###^, p*<*0.001 compared to AAP 2 mM (A2). (**C**) NAC (100* µ*M) as well as TTD (0.1* µ*M), by reducing free radical production, markedly reduce AAP-mediated toxicity on rat cortical neurons. Cortical neurons were incubated with vehicle (DMSO 1‰; V) or AAP 2 mM (A2) alone or in the presence of NAC 100 µM (A2+NAC) or TTD 0.1 µM (A2+TTD) for 24 h and percentage of LDH released to the culture medium was determined. Data represent the mean ± SEM of 9 independent experiments. ***, p*<*0.001 as compared to AAP 2 mM-treated cells.

N-Acetyl cysteine (NAC) increases intracellular glutathione and is the drug of choice to treat AAP-induced liver toxicity [19], [20]. Since the mechanism of action of this antioxidant is mainly related to maintaining GSH levels in most mammalian cells [11], [21], we focused our interest on the effect of AAP on GSH levels in cortical neurons. Quantification of total GSH content, in vehicle- and AAP-treated cells for 18 h, showed that AAP reduced GSH levels to 50% of control values in a concentration-dependent manner (Figure 5A). This toxic effect was prevented either by the presence of 100 µM NAC or by the CYP2E1 inhibitor disulfiram (TTD; 0.1 µM), which restored GSH content to vehicle-treated levels (Figure 5A).

It has also been described that a decrease in GSH levels might be related to an increase in reactive oxygen species (ROS) production [11], [21] that can activate different death signalling pathways in neuronal tissues [22]. We thus decided to study ROS production in AAP-treated cortical neurons. Cells were treated with AAP at different concentrations for 18 hours and then the rate of ROS production was monitored using the fluorescent dye CM-H_2_-DCFDA. The results showed that AAP increased the rate of ROS production in a concentration-dependent manner (Figure 5B). To determine if this increase in ROS production was related to AAP-induced CYP2E1 activity and caused a decrease in GSH levels, we studied the effect of NAC and of the CYP2E1 inhibitor TTD. Pre-treatment of cortical neurons with NAC (100 µM) or TTD (0.1 µM) markedly reduced AAP(2 mM)-induced ROS generation (Figure 5B). These results suggest that ROS generation, through decreasing GSH levels, plays a key role in the mechanism by which AAP induces rat cortical neuronal death.

To analyse whether metabolism through CYP2E1 was involved in the AAP toxicity mechanism, we determined the effect of TTD on AAP-induced neuronal death. The results showed that TTD markedly prevented AAP toxic effect on neuronal viability, decreasing cellular death to an extent similar to that obtained with NAC treatment (Figure 5C). These results suggest that chemically reactive AAP metabolites and GSH depletion play a central role in the AAP neurotoxicity mechanism.

### AAP metabolism by CYP2E1 plays a key role in neuronal toxicity

**Figure 6:**
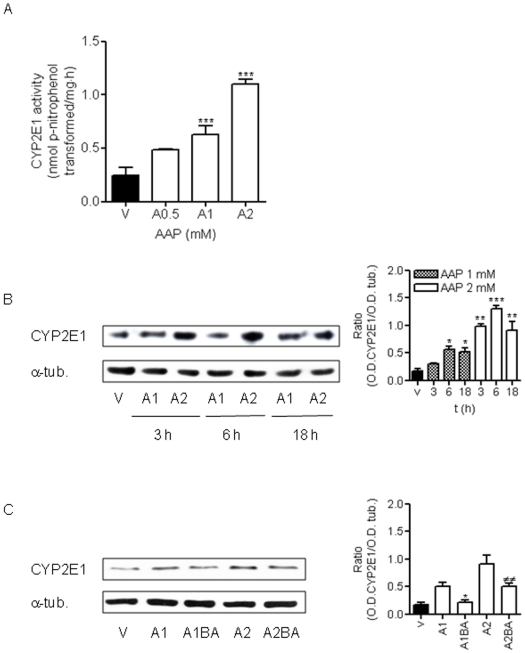
Free radicals generated from AAP metabolism, through CYP2E1 activity, play a central role in AAP-induced neuronal death, in a mechanism dependent on mitochondrial permeability transition. (**A**) AAP increases CYP2E1 activity in a concentration-dependent manner in rat cortical neurons. Cells were treated with vehicle (DMSO 1‰; V) or AAP 0.5 (A0.5), 1 (A1) and 2 mM (A2)) for 18 h and total lysates were obtained. CYP2E1 activity was quantified as nmol p-nitrophenol transformed per milligram of protein in total lysates. Data represent the mean ± SEM of 9 independent experiments. *** p*<*0.001 as compared to vehicle-treated cells (V). (**B**) AAP increases CYP2E1 protein levels in a time- and concentration-dependent manner. Left. CYP2E1 expression detected in total lysates obtained from cells treated with vehicle (DMSO 1‰; V) or AAP at 1 mM (A1) or 2 mM (A2) for 3, 6 and 18 h. α-Tubulin (α-tub) was used as protein loading control. Right. Densitometric analysis of CYP2E1 related to α-tubulin (α-tub.) protein levels detected in total lysates. Data are expressed as mean ± SEM of 3 independent experiments. *p<0.05; **p<0.01; ***p<0.001 compared to vehicle-treated cells. (**C**) Bongkrekic (BA), by inhibiting mitochondrial permeability transition, prevents AAP-induced CYP2E1 induction. Left. CYP2E1 expression detected in total lysates obtained from cells treated with vehicle (DMSO 1‰; V) or AAP 1 mM () and 2 mM in the absence (A1; A2) and the presence of BA 20 µM (A1BA; A2BA) for 18 h. α-Tubulin (α-tub.) was used as protein loading control. Right. Densitometric analysis of CYP2E1 related to α-tubulin (α-tub.) protein levels detected in total lysates. Data are expressed as mean ± SEM of 3 independent experiments. *p<0.05 as compared to A1-treated cells; ^##^, p*<*0.01 as compared to A2-treated cells.

It is well-known that AAP is mainly metabolised via conjugation with glucuronic acid and sulphate and then excreted, but a small fraction of AAP is metabolised by the cytochrome P-450 isoform CYP2E1, forming a highly chemically reactive metabolite, NAPQI [8], [23]. For this reason, we decided to study CYP2E1 enzymatic activity in rat cortical neurons. In vehicle-treated neurons, low levels of CYP2E1 activity were detected (Figure 6A). However, AAP treatment for 18 h increased CYP2E1 activity in a concentration-dependent manner, indicating that this pathway for the metabolism of AAP was increased in cortical neurons (Figure 6A). In addition, AAP induced an increase in CYP2E1 expression as indicated by the rise in CYP2E1 protein levels (Figure 6B). Blocking the mitochondrial permeability transition using bongkrekic acid prevented an AAP-mediated increase in CYP2E1 expression (Figure 6C).

### *In vivo* toxicity of AAP on cortical neurons

Next, we explored whether AAP concentrations that produced neuronal death *in vitro* could be achieved in the CSF of rats following AAP administration, and whether it would cause neuronal death. To fulfil this aim, we injected AAP (250 and 500 mg/Kg) i.p. to rats and measured AAP plasma and CSF levels at different times after injection.

**Figure 7:**
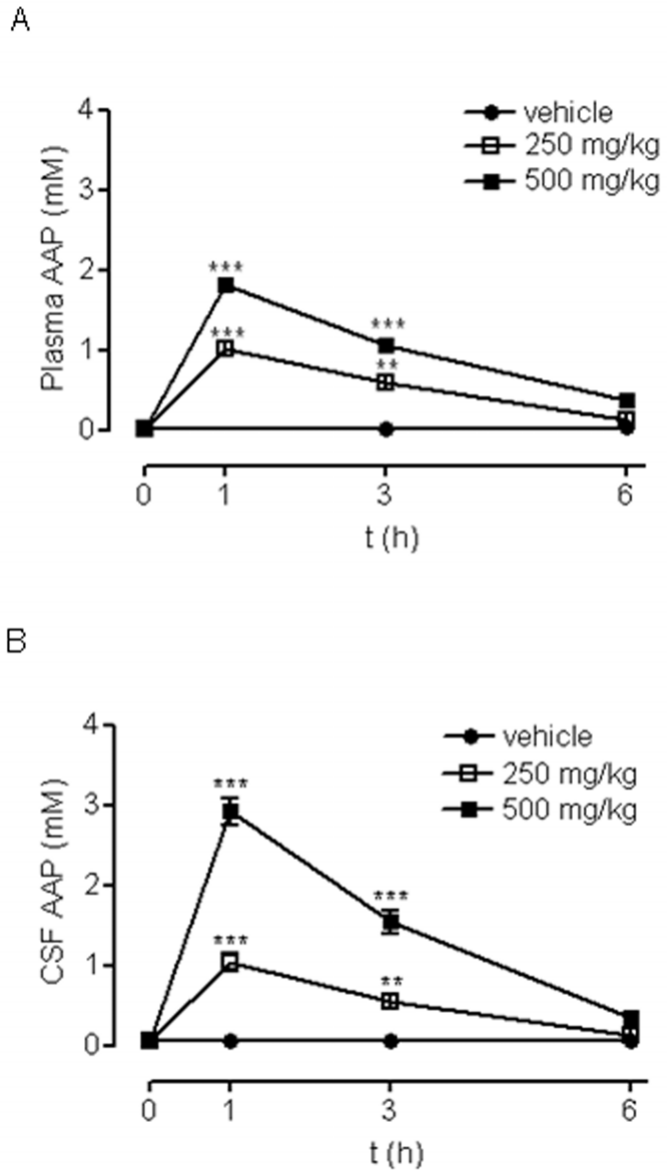
Time-course of rat plasma and cerebrospinal fluid (CSF) AAP levels. (**A**) Time-course of AAP plasma levels measured after intraperitoneal administration of vehicle (H_2_O:PEG, 1∶1; •), AAP 250 mg/kg (□) and 500 mg/kg (▪). Data represent mean ± SEM of 6 animals. **p<0.01; ***p<0.001 as compared to vehicle-treated cells. (**B**) Time-course of AAP CSF levels measured after intraperitoneal administration of vehicle (H_2_O:PEG, 1∶1; •), AAP 250 mg/kg (□) and 500 mg/kg (▪). Data represent mean ± SEM of 6 animals. **, p<0.01; *** p<0.001 as compared to vehicle treated cells.

**Figure 8:**
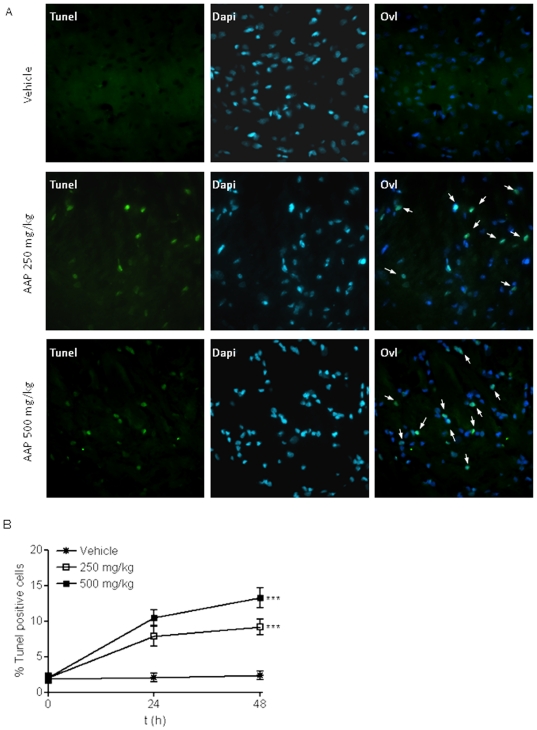
Acetaminophen toxicity in vivo. A. Brain cortex tissue slices (5 µM thick) from vehicle (V; H_2_O:PEG, 1∶1), AAP (250 mg/Kg i.p.) and AAP (500 mg/Kg i.p) injected animals obtained 24 h post-injection, were double-stained with Dapi to identify nuclei, apoptotic neurons were labelled using TUNEL as indicated in Material and Methods. An overlay of both signals (Ovl) is also presented. Arrows indicate cells showing an overlay of TUNEL and Dapi signals. The figure shows a representative experiment that was repeated 3 times with similar results. **B.** Quantification of damaged neurons. Data represent the percentage of TUNEL positive cells related to total Dapi stained cells. For quantification, a total of 12 image fields from 2 different animals were used for each condition. The average number of cells per field was 79.1±2.8 (mean ± SEM). For each field, the percentage of TUNEL positive neurons was determined. The total number of neurons (100% of the y axis) counted for each experimental condition was: 990 for vehicle; 1050 for AAP (250 mg/Kg)-treated rats and 920 for AAP (500 mg/kg)-treated rats. Data represent mean ± SEM of the percentage of TUNEL positive cells in 12 image fields from 2 different animals for each condition. ***p<0.001 as compared to vehicle treated animals.

We found that one hour after injection, AAP plasma levels reached 1 and 2 mM for i.p. injections of 250 and 500 mg/Kg respectively. These levels remained elevated at 3 h after injection (0.6 and 1.1 mM respectively), decreasing to basal levels 6 h after injection (Figure 7A). Cerebrospinal fluid levels followed this pattern but at slightly higher concentrations (1 and 3 mM for i.p. injections of 250 and 500 mg/Kg) (Figure 7B), according to the well-known ability of AAP to cross blood-brain barrier [24]. These data show that, at least for 3 h, AAP levels that were toxic to rat cortical neurons *in vitro* were achieved in rat CSF following AAP i.p. administration. When the effect of the AAP i.p. injection (250 mg/Kg and 500 mg/Kg) on rat brain cortex neurons was explored, an increase in the number of TUNEL positive neurons was observed, suggesting an AAP-mediated time- and concentration-dependent neuronal toxicity in that area of the brain (Figure 8)

**Figure 9:**
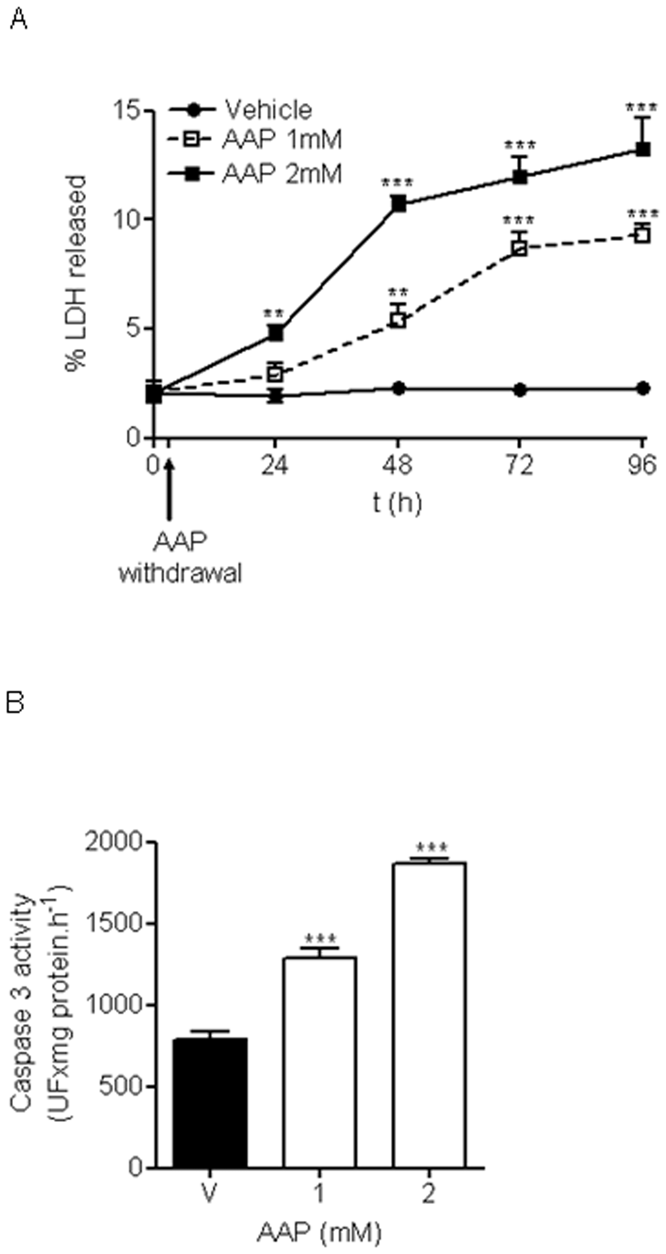
Toxicity of transient exposure to AAP. **A.** Cortical neurons were incubated with vehicle (H_2_O:PEG, 1∶1; •), AAP 1 mM (□) and AAP 2 mM (▪) and 3 h later, the medium containing the drug was removed (AAP withdrawal) and replaced with medium lacking the drug, mimicking what happens *in vivo*. The percentage of LDH released was measured to study cumulative toxicity at different times. Data represent mean ± SEM of 9 independent experiments. **p<0.01; *** p<0.001 as compared to vehicle treated cells. **B**. Same protocol as in A, but caspase 3 activity was determined at 18 h after treatment. Data represent mean ± SEM of 9 independent experiments. ***p<0.001 as compared to vehicle-treated cells (v).

Next, we explored whether a transient exposure to AAP for 3 h, followed by drug withdrawal, was enough to induce cellular death *in vitro*. This experimental protocol would mimic what happens with CSF AAP levels *in vivo* following AAP i.p. injection. We found that a single 3-h exposure to AAP (1 and 2 mM) induced a small but progressive neuronal death *in vitro* that affected as many as 10% of the neurons 96 h after AAP treatment (Figure 9A). Accordingly, caspase 3 activity was found to increase in cortical neurons transiently exposed to AAP (1 and 2 mM) (Fig. 9B).

## Discussion

Our experiments show that AAP, a drug that is widely used (more than 70% of the population in western countries has taken at least once AAP and a relevant percentage takes the drug chronically) as a mild pain reliever and antithermic [3], has direct neurotoxic effects on rat brain neurons both *in vitro* and *in vivo*. Moreover, these toxic effects are produced *in vitro* at concentrations that are achieved transiently (for about 3 h) in rat CSF following AAP i.p. injection. It is important to note that *in vitro* transient exposure (3 h) of cultured rat cortical neurons to AAP concentrations, similar to those achieved in CSF following AAP i.p. injection (0.5 to 2 mM), produces an increase in caspase 3 activity that leads to a small but progressive neuronal death that increases with time. This toxic effect seems to be mediated by AAP metabolism by the cytochrome P-450 CYP2E1 isoform that generates a highly reactive metabolite NAPQI. This highly reactive compound is conjugated with GSH, producing a decrease in GSH levels in neurons that leads to neuronal death. Accordingly, co-treatment of neurons in culture with NAC, which restores GSH levels, prevents neuronal death.

We have found that AAP, at the doses used (above 1 mM *in vitro* and 250 mg/Kg *in vivo*), is neurotoxic, both in vivo and in vitro, to rat cortical neurons. This is the first time that this toxic AAP effect on neurons has been described. It had previously been described that AAP 1 mM did not affect the total number of mesencephalic neurons for 48 h [25], whereas we have detected a loss in cortical neuronal viability at 24 h. Differences with our results might be explained by the different kind of neurons studied. On the other hand, Bisaglia et al. have shown that AAP (100 µM) protects hippocampal neurons and PC12 cultures from amyloid beta-peptides [26]. Moreover, AAP (100 µM) pre-treatment also prevented menadione-induced neurotoxicity [27] and AAP (100 mg/kg) protected against oxidative neurotoxicity *in vivo* at 4 h after its administration every hour for 3 hours [28]. Locke et al. have shown that AAP (100 µM) protected *C. elegans* dopamine neurons from 6-OHDA exposure [29]. However, when AAP was raised to 1 mM, the protection was only apparent soon after the application and was lost when higher concentrations (2 and 9.2 mM) were used. Moreover, it has been recently shown that intraperitoneal administration of AAP (5–100 mg/kg) seems to have protective effects on oxidative stress-induced brain toxicity by inhibiting free radical production [30]. However, no beneficial effects were observed when AAP was administered at doses of 200 or 500 mg/Kg. In contrast,On the other hand, 1 mM AAP was significantly toxic to stage I HUCB-NSCs, in agreement with our observations [31].

To summarise, it seems that AAP concentrations up to 100 µM or AAP doses up to 100 mg/Kg prevent ROS production and citotoxicity, but higher doses do not. Furthermore, several recent studies have shown the protective effects of antioxidants on hepatic failure induced by AAP overdose [32]–[34], indicating that high AAP concentrations, far from protecting cells from ROS toxicity, actually contribute to ROS production. In agreement with these data, we have shown that AAP increases ROS production in a concentration-dependent manner 18 h after treatment.

Acetaminophen is mainly metabolised, in the liver, via conjugation by sulphotransferase and UDP-glucuronosyltransferase and then excreted, but a small fraction of it is metabolised by CYP2E1 [7], [35]. This P-450 isoenzyme is very abundant in the liver [36] and is also expressed in the brain [10], although at much lower levels, where it seems to play an important role in metabolizing some compounds like AAP and ethanol. CYP2E1 is the most important P-450 isoenzyme involved in AAP metabolism, although other isoforms like CYP1A2 or CYP3A might also be involved [37]. It has long been known from the work of the Brodie group [38], [39] that induction of P-450 enhances AAP-mediated liver toxicity [36]. Acetaminophen metabolism by CYP2E1 produces a chemically reactive metabolite, NAPQI, that might bind to sulfhydryl groups in cellular proteins, including mitochondrial proteins, inducing oxidative stress and leading to cellular damage and death [40], [41]. Generally, NAPQI reacts with GSH to form a non-toxic conjugate that will be excreted [42], [43]. Excess NAPQI production, due to increased AAP levels and substrate-induced CYP2E1 stabilisation [36], leads to a reduced GSH content in cortical neurons in culture in a concentration -dependent manner.

Rats exposed to APP have shown unchanged levels of AAP in the brain and kidneys, levels similar to those found in other tissues [6]. These authors discarded a possible toxic effect of AAP in those tissues due to the low activity of the cytochrome P-450 system, as determined by the lower covalent binding in the brain and kidneys when compared to the liver, but they did not study the effect of AAP on renal or brain cells viability directly [6]. Later studies showed that AAP can induce renal tubular damage and acute renal failure in the absence of liver injury by a mechanism related to NAPQI production through cytochrome P-450 activation and glutathione depletion, which would lead to NAPQI binding to cellular proteins [44]–[47], despite the low levels of AAP-glutathione conjugate found in kidney by Fisher and co-workers. Our experiments show that cortical neurons not only express the 2E1 isoform of cytochrome P-450, but also that AAP enhances its levels and activity after 18 h of treatment. Since AAP is found unaltered in the brain and AAP increases CYP2E1 expression and activity, AAP could induce cortical neuron apoptosis due to increased local NAPQI production through CYP2E1 enzymatic activity. In addition, TTD, an inhibitor of the 2E1isoform of cytochrome P-450, significantly reduced AAP-induced neuronal death, suggesting a role for this enzymatic activity in the mechanism involved in AAP-mediated neuronal death. Moreover, an increase in AAP-mediated ROS levels could also contribute to neuronal damage. The combination of NAPQI, which could damage mitochondria, and ROS, contributing to cyt C release, would activate the intrinsic death pathway leading to caspase 3 activation and apoptotic death.

GSH is a thiol-containing coenzyme that plays a relevant role in several redox reactions in the body, including the maintenance of the thiol properties of reduced coenzyme A, cysteine and vitamins C and E. GSH is the main cellular antioxidant, and is found at concentrations of between 1 and 10 mM in most mammalian cells, including neurons. Its depletion could induce accumulation of NAPQI, which might mediate the toxic effect observed with AAP treatment through the mechanisms described above. According to this proposed mechanism, NAC, a drug that increases intracellular glutathione levels, prevents AAP-induced neuronal death. The mechanism involved is similar to that observed in the liver, but this is the first time that it has been described in neuronal cells, both *in vitro* and *in vivo*, following AAP treatment.

Although it is generally assumed that LDH efflux *in vivo* represents necrotic cell death, in cell culture, quantification of LDH, rather than necrotic death, represents cellular death in general. In necrotic death *in vivo*, cytoplasmic membrane lyses and intracellular content is released, resulting in inflammation [48], whereas in apoptosis, the cellular contents are safely sealed within the dying cells until phagocytosis intervenes. However, in culture cells the phagocytic step after apoptosis does not occur and apoptotic cells undergo secondary necrosis, releasing their contents into the surrounding medium [49]. Acetaminophen-induced neuronal death exhibits the hallmarks of apoptotic death, such as DNA fragmentation and degradation, showing a ladder-type image (Figure 2A), suggesting activation of the mitochondrial pathway and ROS production in AAP-mediated neuronal toxicity. Moreover, the observed cyt C release, ROS generation and activation of caspase 3 in response to AAP support this point. In addition, mitochondrial permeability transition seems to play a central role in this mechanism since bongkrekic acid, an inhibitor of mitochondrial adenine nucleotide translocase [50], prevents both caspase 3 activation and the loss of neuronal viability.

These neurotoxic AAP *in vitro* actions can be also found *in vivo*. It is known that AAP crosses the blood-brain barrier both in humans and rodents [24], but it is important to note that the data presented here show that, following i.p. AAP injection, there is an increase in CSF drug levels that reaches transiently, for about 3 hours, the AAP concentrations that produce rat cortical neuronal death *in vitro*. Accordingly, an increase in the number of TUNEL positive neurons, suggestive of neuronal death, can be observed in rat brain cortex following AAP i.p. injection. On the other hand, transient exposure of cultured cortical neurons to the same concentrations achieved in CSF following AAP i.p. injection induces a delayed neuronal death.

It has been previously described that AAP overdose is the most common cause of acute liver failure (ALF) in western countries [51], [52]. One of the main causes of morbidity and mortality in ALF is encephalopathy [53]. Encephalopathy has been related to the “glutamine hypothesis” wherein detoxification of ammonia by astrocytes leads to conversion of glutamate to glutamine, which can increase tissue osmolarity and cause oedema [54], [55]. Alternatively, cerebral oedema may also develop from failure of intracellular vascular autoregulation with resultant increases in brain water and brain volume [54], [56]. Despite these indirect actions, no evidence of a direct action of AAP on neuron viability has been described.

Furthermore, it was recently found that AAP overdose (400 mg/kg, i.p.) reduces creatine kinase activity (CK) in the cerebellum and hippocampus but not in other brain areas [57]. CK catalyses ATP regeneration from ADP and decreased activity has been associated with neurodegenerative pathways [58], [59]. It has been postulated that CK inhibition is probably involved in the pathogenesis of hepatic encephalopathy [57]. Taken together, these results suggest that the neurotoxic action of AAP described here is relevant *in vivo*, in the rat, and that it might happen in other mammalian species including humans.

In summary, our data indicate that, in rats, AAP produces neuronal death of cortical neurons, both *in vivo* and *in vitro*, at the same concentration range. This neurotoxic action seems to be mediated by activation of the neuronal cytochrome P450 isoform CYP2E1 and generation of the toxic metabolite NAPQI, which exhausts GSH levels leading to neuronal death through mitochondrial–mediated mechanisms. This newly described effect of a very widely used drug like AAP suggests that this neurotoxic effect might contribute to AAP overdose—mediated toxicity. Further studies should be conducted to determine if this neurotoxic action of AAP is a drug-class effect shared by other NSAIDs or by COX-inhibiting drugs.

## Materials and Methods

### Animal handling. Ethics statement

All animals were treated and killed in accordance with European Union (2003/65/CE) guidelines on the use of laboratory animals. Protocol for animal handling and sacrifice was approved by the Comité de Ética de Experimentación Animal of the Universidad de Castilla-La Mancha.

### Cell culture

Primary cultures of brain cortical neurons were essentially prepared as described previously [60]. After isolation, the cells were resuspended in serum-free Neurobasal medium supplemented with B27, containing 2 mM L-glutamine, penicillin (20 units/ml) and streptomycin (5 µg/ml), and plated on poly-L-lysine-coated 24- or 6-well culture plates or on poly-L-lysine-coated glass coverslips. The combination of Neurobasal, B27 and the lack of serum minimised glial proliferation. The culture contained around 95% pure neurons when determined by glial fibrillary acidic protein (GFAP) and neuronal-specific (NeuN) antigen immunostaining (data not shown). Cells were maintained at 37°C in a saturated humidity atmosphere containing 95% air and 5% CO_2_ and used for experiments after 7–10 days *in vitro* (DIV).

### Viability experiments

After 7-10 DIV neurons were treated with vehicle (DMSO 1‰) or AAP at different concentrations for the times indicated. Supernatants were then collected and cells were washed with PBS and lysed with 0.9% Triton X-100 (v/v) in saline. LDH activity, used as an index of neuronal death, released to culture media, as well as LDH present in lysates, was measured spectrophotometrically at 490 nm on a 96-well plate reader using the Cytotox 96 Kit (Promega) as previously described [61]. Mortality was expressed as a percentage of LDH released.

### Hoescht 33342 staining

Cells were plated on poly-L-lysine-coated glass coverslips and, after 7-10 DIV, treated with vehicle, AAP 0.5 and 1 mM for 24 h. After the incubation period, the cells were loaded with Hoescht 33342 by incubation (1 µM for 5 min at 37°C) in Krebs-Henseleit solution (with the following composition in millimoles/Liter: NaCl 140, KCl 5.9, MgCl_2_ 1.2, HEPES 15, glucose 10, CaCl_2_ 2.5, pH 7.4). The cells were then washed twice with Krebs-Henseleit solution and fluorescence was observed using a 350-nm excitation filter and a 450-nm emission filter in a Nikon Diaphot inverted microscope equipped with a 75W Xenon lamp and a Nikon 40X, 1.3 numerical aperture, epifluorescence oil immersion objective. Images were taken with a CCD camera using commercial software (Metamorph, Universal Imaging Corporation, Silicon Valley, CA, USA).

### DNA fragmentation analysis

DNA fragmentation was studied as previously described [62]. The cells were plated on poly-L-lysine-coated 6-well culture plates and, after 7-10 DIV, treated with vehicle (DMSO 1‰), staurosporine (500 nM) or AAP at different concentrations. Twenty four hours later, the cells were collected by scraping and centrifuged at 800×*g* for 10 min. Pellets were washed twice with PBS-MgCl_2_ 5 mM and then resuspended in Lysis buffer (50 mM Tris-HCl, 50 mM NaCl, 10 mM EDTA, 0.5% SDS pH 7.4) containing 0.125% (w/v) proteinase K and maintained at 50°C overnight. After centrifugation at 10,000×*g* for 10 min at 4°C, fragmented DNA in the supernatant was extracted by adding a mixture of phenol/chloroform/isoamyl alcohol (24∶24∶1) and centrifuged at 10,000×g for 10 min at 4°C. Fragmented DNA in the aqueous phase was precipitated by adding 3 M sodium acetate and 800* µ*l of absolute ethanol and then isolated by centrifugation at 10,000×*g* for 20 min. The DNA pellet was dissolved in 25* µ*l of a 10 mM Tris-HCl, pH 7.4 solution containing 1 mM EDTA. The DNA samples were subjected to electrophoresis on 1.5% agarose gel and then observed under UV light after staining with ethidium bromide.

### Subcellular fractionation

Cells were plated in poly-L-lysine-coated 6-well culture plates and, after 7-10 DIV, treated with vehicle (DMSO 1‰) or AAP at 0.5, 1 and 2 mM for 24 hours. Afterwards, the cells were washed twice with PBS, scraped and collected by centrifugation at 1,500×*g* for 10 min. The cell pellets were resuspended in 200 µl of extraction buffer (250 mM sucrose, 50 mM Tris-HCl, 1 mM EGTA, 2.5 mM EDTA, 50 µM Na_3_VO_4_, 1 mM DTT, 0.1 mM PMSF, 40 µg/ml aprotinine, 20 µg/ml leupeptine; pH 7.4) and homogenised with a pellet pestle (15 strokes) and, after 15 min on ice, centrifuged at 900×g for 5 min at 4°C. The pellets, containing nuclei and intact cells, were discarded and the supernatants were centrifuged at 20,000×*g* for 30 min at 4°C. The supernatants, i.e. cytosolic fractions, were removed and stored at −80°C until analysed by gel electrophoresis. Pellets containing mitochondria were resuspended in 50 µl of extraction buffer, homogenised with a pestle (5 strokes) and then centrifuged at 20,000×*g* for 60 min at 4°C. The supernatants, i.e. mitochondrial fractions, were removed and analysed by gel electrophoresis.

To obtain whole lysates, cells were plated on poly-L lysine coated 6-well culture plates and, after 7-10 DIV, treated with vehicle (DMSO 1‰) or AAP at 1 and 2 mM for 3, 6 and 18 h, or with vehicle (DMSO 1‰) or AAP at 1 and 2 mM in the presence or absence of bongkrekic acid (BA) 20 µM for 18 h. Afterwards, the cells were washed twice with PBS and lysed in lysis buffer containing 25 mM Tris-HCl, 25 mM NaCl, 5 mM DTT, 0.1% deoxycholic acid and 1% Triton x-100. Extracts were then centrifuged at 5,000×*g* for 10 min at 4°C and supernatants (total lysates) were removed and stored at −80°C until analysed by gel electrophoresis.

### Western Blot Analysis

Western blot analysis was performed as previously described [63]. Protein samples (30 µg) were loaded on 15% PAGE-SDS and transferred onto nitrocellulose membranes. Membranes were blocked in PBS-Tween 20 (0.1%) containing 5% non-fat dry milk and 0.1% BSA for 1 h at 4°C and incubated with CYP2E1 polyclonal antibody (1∶1,000) (Abcam, Cambrige, UK), anti-cytochrome c polyclonal antibody (1∶1,000) (BD Biosciences, Madrid, Spain), anti-α-tubulin polyclonal antibody (1∶2,000) (Merck Chemicals Ltd., Barcelona, Spain) or anti-OxPhos Complex IV subunit IV (COX-IV) monoclonal antibody (1∶1,000) (Cell Signalling Technology, Barcelona, Spain) overnight at 4°C. Afterwards, the blots were washed with PBS-Tween 20 (0.1%) and incubated with Horse Rabbit Peroxidase conjugated (HRP)-anti-mouse IgG (1∶10,000) (Jackson ImmunoResearch Laboratories, Madrid, Spain) for 2 h at 4°C. Immunoreactive bands were observed using an enhanced chemiluminiscence system (ECL; GE Healthcare, Barcelona, Spain).

Densitometric analysis of immunoreactive bands was performed by using the ImageQuant 5.2 program (GE Healthcare, Barcelona, Spain). The results were expressed as the ratio of optical densities for CYP2E1 (O.D. CYP2E1), cytochrome c (O.D. cyt. C) and α-tubulin (O.D. α-tub) or for COX-IV (O.D. COX-IV).

### Caspase 3 activity

Caspase 3 activity was studied as previously described [64]. Cells were plated in poly-L-lysine-coated 6-well culture plates and, after 7-10 DIV, treated with vehicle (DMSO 1‰) or AAP 2 mM for 3, 6, 18 and 24 h. Afterwards, the cells were washed twice with cold PBS and lysed in Lysis buffer containing 100 mM Hepes pH 7.4, 5 mM DTT, 5 mM EGTA, 0.04% Nonidet P-40, and 20% glycerol. Extracts were then centrifuged at 5,000×*g* for 10 min at 4°C. Cell extracts (40* µ*g of protein) were incubated in reaction buffer (25 mM Hepes, 10% sucrose, 0.1% CHAPS, 10 mM DTT) containing 50* µ*M fluorescent substrate Z-DEVD-AFC at 37°C for 1 h. Cleavage of the AFC fluorophore was determined in a spectrofluorometer at an excitation wavelength of 400 nm and fluorescence was detected at an emission wavelength of 505 nm. Caspase 3 activity was expressed as units of fluorescence/mg of protein/h.

### Glutathione measurement

Glutathione levels were determined as previously described [22]. Briefly, cells were plated in poly-L-lysine-coated 6-well culture plates and, after 7-10 DIV, treated with vehicle (DMSO 1‰) or AAP at 1 and 2 mM for 18 h. In another set of experiments, cells plated in 6-well plates maintained for 7-10 DIV were treated with vehicle (DMSO 1‰) or AAP alone or in combination with N-acetylcysteine (NAC) or disulfiram (TTD) for 18 h. Then, the cells were washed twice with cold PBS and scraped in 1 ml of PBS. The cells collected were counted and then centrifuged at 1,500×*g* for 10 min and the pellet was resuspended in 5-sulphosalicylic acid (3.33%) containing 0.25 mM EDTA to prevent glutathione (GSH) oxidation and to inhibit GSH-utilizing enzymes. The tubes were frozen and thawed three times to break the cells and release GSH. The lysates were then centrifuged at 10,000×*g* for 5 min at 4°C and the supernatants transferred to eppendorf tubes kept on dry ice until assayed for glutathione content. Glutathione measurements were performed as previously described [65]. GSH reacts non-enzymatically with 5,5′-dithiobis-(2-nitrobenzoic acid) to generate oxidised glutathione (GSSG) and the highly coloured 5-thio-2-nitrobenzoic acid (peak absorbance 420 nm); the GSSG that forms is back reduced to GSH by glutathione reductase coupled to NADPH oxidation. In this cycling assay, the rate of colour formation is linear in time and the slope is directly proportional to total glutathione (GSH + GSSG; GSt) concentration. Standards and cell lysates, assayed in triplicate, were incubated in reaction buffer (0.125 M phosphate buffer pH 7.5, containing 0.21 mM NADPH, 0.6 mM DTNB, 6.3 mM EDTA and 2 U glutathione reductase) at 37°C and absorbance was measured every 15 s over 1 min at 420 nm. Concentrations obtained were normalised by the number of living cells after treatments. Although NAC contains a sulfhydryl group that might react with 5,5′-dithiobis-(2-nitrobenzoic acid) (DTNB) - the color-generating reagent in the assay mixture - the enzymatic cycling mediated by glutathione reductase provides the required assay specificity to measure glutathione, and prevents the interference from other compounds having the sulfhydryl group [66]. In addition, GSH levels measured in cortical neurons treated with vehicle or only with TTD showed no differences in GSH content between vehicle- and TTD-treated neurons, confirming that even though TTD is a substrate for glutathione reductase [67] and is reduced to diethylsithiocarbamate, the method used was specifically for measuring GSH.

### Cytochrome P450 2E1 isoform activity

Cells were plated in poly-L-lysine-coated 6-well culture plates and, after 7 DIV cells, treated with vehicle (DMSO 1‰) or AAP at 0.5, 1 and 2 mM for 18 h. Then, the cells were washed twice with cold PBS and scraped in 1 ml of PBS and collected by centrifugation at 1,500×*g* for 10 min. Cell pellets were resuspended in 200* µ*l of extraction buffer (100 mM KH_2_PO_4_, pH 7.4) and homogenised with a pellet pestle (Sigma) (30 strokes) and then centrifuged at 10,000×*g* for 30 min at 4°C. Cell lysates (60 µg) were incubated in reaction buffer (50 mM Tris buffer, pH 7.4, containing 5 mM MgCl_2_, 0.5 mM NADPH and 0.5 mM p-nitrophenol) at 37°C for 30 min. The reaction was stopped by the addition of 0.6 N perchloric acid, and after precipitation of proteins by centrifugation at 10,000×g for one minute at 4°C, supernatants were mixed with 10 N NaOH. The absorbance was measured at 535 nM [68]. Cytochrome P450 2E1 isoform (CYP2E1) activity was expressed as nmoles of p-nitrophenol transformed/mg of protein/h. The fact that CYP2E1 is constitutively expressed in the brain and is induced by different drugs, including AAP, [69], [70] and that AAP is mainly metabolised by the 2E1 isoform of cytochrome P-450 [7] suggests that the P-450 enzymatic activity observed in treated neurons corresponds to the CYP2E1isoform.

### Free-radical production

Cells were plated in poly-L-lysine-coated glass coverslips and, after 7 DIV, treated with AAP 0.5, 1 and 2 mM, alone or in combination with either NAC or TD, for 18 h. To monitor free-radical production, the cells were loaded by incubation with CM-H_2_DCFDA (Molecular Probes; Barcelona, Spain) (10 µM for 30 min at 37°C) in Krebs-Henseleit solution as described previously [71]. Images were taken with a CCD camera and analysed using commercial software (Universal Imaging Corporation, Silicon Valley, CA, USA). Background was subtracted and fluorescence was recorded using a 535-nm excitation filter and a 635-nm emission filter. Frames were recorded every 15 s over a 10-min period. Fluorescence data for each condition were fitted to the equation y = a+bx, and the slope **b** was taken as an index of the rate of superoxide production [22].

### Quantification of AAP concentration in LCR and plasma obtained from AAP-injected rats

Female Sprague-Dawley rats weighing 240–260 g were divided into groups (6 to 8 animals each group) and injected intraperitoneally with vehicle (H_2_O:polyehylene glycol (PEG) 1∶1) or AAP at the doses of 250 or 500 mg/kg in 1 ml of (H_2_O:PEG 1∶1). After 1, 3 or 6 h, the animals were anesthetised with halothane and 1 ml of blood was obtained by intracardiac puncture. The blood was mixed with 1% sodium citrate in saline and centrifuged (4,000×g, 10 min, 4°C) to obtain the plasma. Similarly, 100 µl of brain cerebrospinal fluid (CSF) was obtained by cisternal punction. Afterwards, the animals were killed by decapitation. Plasma and brain cerebrospinal fluid samples were frozen at −80°C until analysed.

Plasma and CSF AAP concentrations were measured using a HPLC method described previously [72].

### Immunohistochemical analysis of in vivo neuronal death

Paraffin-embedded rat frontal cortex sections (5 µm thick) were deparaffinised, rehydrated, and subjected to high-temperature antigen retrieval in 10 mmol/L sodium citrate buffer, pH 6. The slices were incubated with 4′, 6 diamidine-2-phenylindole (DAPI; 5 µg/ml) to identify nuclei. Apoptotic cells were identified by the TUNEL technique (*In Situ* Cell Death Detection kit, fluorescein labelled. Roche Diagnostics, Mannheim, Germany), used according to the manufacturer's instructions. The sections were analysed using a Nikon inverted microscope (Nikon Eclipse TE-2000-S, Birlingam, CA, USA) equipped with a 150 W Xenon lamp and a 100X, 1.3 numerical aperture, epifluorescence oil immersion objective. The excitation wavelength was selected using a Life Technologies monochromator (Omega Optical Inc, Brattleboro, VT, USA) and the emission wavelength using a filter wheel (Sutter Instruments, Novato, CA, USA). Images were taken using a digital camera (ORCA II, Hamamatsu, Shizouka, Japan). The data were analysed using commercial software (Metamorph, Universal Imaging Corporation, Silicon Valley, CA, USA). The following wavelengths were used for each fluorescent dye: DAPI, ex: 350 nm, em: 450 nm. TUNEL, ex: 480 nm, em: 520 nm. Six randomly selected image fields from the cortex of two different animals were analysed for each experimental condition.

### Analysis

Data are expressed as mean ± SEM. Statistical analyses were carried out using the one-way analysis of variance (ANOVA) and the *a posteriori* Bonferroni's *t*-test for multiple comparisons. *P* values of less than 0.05 were considered significant (*p<0.05, **p<0.01, ***p<0.001). Statistical results are reported in the figure legends.

### Drugs and Chemicals

The BCA protein assay kit was from Pierce Biotechnology Inc. (Illinois, USA). The Cytotox 96 kit was from Promega Biotech Iberica S.L. (Madrid, Spain). Z-DEVD-AFC was from Calbiochem (Madrid, Spain). CM-H_2_DCFDA was from Molecular Probes Inc. (Barcelona, Spain). All other reagents were obtained from Sigma-Aldrich (Madrid, Spain).
